# Vehicle Localization Method in Complex SAR Images Based on Feature Reconstruction and Aggregation

**DOI:** 10.3390/s24206746

**Published:** 2024-10-20

**Authors:** Jinwei Han, Lihong Kang, Jing Tian, Mingyong Jiang, Ningbo Guo

**Affiliations:** 1 Graduate School, Space Engineering University, Beijing 101416, China; jinwei_han@hgd.edu.cn (J.H.);; 2 Beijing Insititute of Remote Sensing Information, Beijing 100192, China

**Keywords:** synthetic aperture radar (SAR) images, vehicle localization, attention mechanism, feature extraction, feature fusion

## Abstract

Due to the small size of vehicle targets, complex background environments, and the discrete scattering characteristics of high-resolution synthetic aperture radar (SAR) images, existing deep learning networks face challenges in extracting high-quality vehicle features from SAR images, which impacts vehicle localization accuracy. To address this issue, this paper proposes a vehicle localization method for SAR images based on feature reconstruction and aggregation with rotating boxes. Specifically, our method first employs a backbone network that integrates the space-channel reconfiguration module (SCRM), which contains spatial and channel attention mechanisms specifically designed for SAR images to extract features. The network then connects a progressive cross-fusion mechanism (PCFM) that effectively combines multi-view features from different feature layers, enhancing the information content of feature maps and improving feature representation quality. Finally, these features containing a large receptive field region and enhanced rich contextual information are input into a rotating box vehicle detection head, which effectively reduces false alarms and missed detections. Experiments on a complex scene SAR image vehicle dataset demonstrate that the proposed method significantly improves vehicle localization accuracy. Our method achieves state-of-the-art performance, which demonstrates the superiority and effectiveness of the proposed method.

## 1. Introduction

Accurate vehicle localization, regardless of weather or time, is crucial for effective management and decision-making and plays a key role in enhancing urban governance and management capabilities. Compared to visible light imaging techniques, a synthetic aperture radar (SAR) is an active microwave imaging system that is not limited by time, light, or other natural conditions. Its ability to achieve all-day, all-weather earth observation has garnered widespread attention. Additionally, when operating at low-frequency bands, SAR possesses some penetration capability, enabling it to detect targets beneath vegetation, camouflage, and other types of cover. These characteristics make SAR highly valuable and versatile in various fields. With advancements in SAR imaging technology, the resolution of SAR images is continually improving, making vehicle detection and localization from high-resolution, large-format images a major research focus.

Vehicle localization methods based on SAR images can be categorized into traditional methods and deep learning methods. Among the traditional SAR image search techniques, the Constant False Alarm Rate (CFAR) method is the most widely used [1,2,3]. This technique relies on the accuracy of background clutter modeling and requires manual feature design to distinguish false alarms, such as those caused by human-made structures or natural features, to achieve a certain level of detection performance when the background is relatively simple. However, a large number of false alarms and missing alarms are often generated for high-resolution, wide-width SAR image scenes under complex background conditions.

In recent years, advancements in deep learning have led to a surge in algorithms that utilize convolutional neural networks (CNNs) for object detection. These methods address many of the limitations of traditional approaches by eliminating the need for accurate background modeling. CNN-based methods excel in feature learning and demonstrate superior localization accuracy and generalization compared to traditional techniques. Duran et al. [4] innovatively combined Region Proposal Networks (RPNs) [5] with a Fast R-CNN [6] to develop a detection network. Their approach demonstrated significantly improved detection performance on the MSTAR and MiniSAR datasets compared to traditional methods. In 2018, Diao et al. [7] found the problem of discrepancies between the predicted candidate region size and the actual bounding box size when applying optical image-based aircraft detection algorithms to SAR images. They first utilized the CFAR algorithm for initial positioning and then applied a Fast R-CNN for the further regression and classification of the regions identified by the CFAR. This multi-step approach improved the algorithm’s multi-scale retrieval accuracy. In 2019, Wang et al. [8] applied the SSD network [9] to SAR images and employed data augmentation and transfer learning to solve the problem of limited amount of SAR images. In the same year, Zhang et al. [10] introduced a high-speed ship detection model based on YOLOv3 [11], which accelerated ship detection by scaling down the backbone network and eliminating redundant layers.

Most SAR image target detection algorithms are based on those designed for optical images, which are improved by combining with traditional SAR image target detection algorithms. However, due to the significant differences in feature dimensions between SAR and optical images, achieving high localization accuracy remains challenging. In optical images, vehicles exhibit rich detail and a degree of rotational invariance. In contrast, SAR images display strong scattering and sparsity in vehicle representations, which are significantly influenced by the microwave incidence angle of ground objects during imaging. Therefore, it is essential to design a specialized vehicle localization network that accounts for the unique characteristics of SAR images.

Many researchers have explored vehicle detection in SAR images. Zou et al. [12] built a vehicle detector SCEDet for small and medium-sized vehicles in SAR images. Their semantic context enhancement module balances depth semantic information with image detail using fewer parameters, and effectively aggregates global context, outperforming other algorithms on the FARAD dataset. Tang et al. [13] built a twin sub-network architecture for the fusion of a two-parameter CFAR and SSD. They designed an interactive channel spatial attention fusion module to fuse the CFAR indicator map and the feature map, which effectively improved the network’s feature extraction of vehicle images, suppressed background clutter, and also alleviated the problem of foreground–background imbalance. Li et al. [14] designed a plug-and-play rectangular invariant rotatable convolution that adaptively adjusts sampling positions for different vehicles to improve the feature representation ability of vehicles. This method can improve the localization performance. Wang et al. [15] proposed an attention feature fusion perception network, which uses a multi-scale semantic attention module to obtain multi-scale and semantic features of the region of interest. The variable multi-scale feature fusion module introduced can effectively fuse different feature information and alleviate the interference of image deformation, yielding a better performance on their dataset. While these methods advance vehicle detection in SAR images, their detecting accuracy remains limited, making practical deployment challenging. The small size of vehicles, their limited proportion in SAR images, and the unclear image outlines all further complicate accurate localization. The horizontal frame detection network has a poor positioning effect on densely arranged vehicles, and there are serious problems, such as missing detection and the positioning accuracy of vehicle targets not reaching the practical level.

To enhance vehicle localization accuracy in SAR images, we present a rotating frame SAR vehicle localization algorithm based on feature reconstruction and aggregation network (FRA-Net). Tailored to the characteristics of SAR and vehicle images, the algorithm introduces a novel attention mechanism and a feature fusion mechanism and utilizes a rotating frame detection head for vehicle position. In the proposed FRA-based vehicle localization algorithm, our main work is as follows:

(1) A space-channel reconstruction module (SCRM) replaces the residual blocks in the CSP-DarkNet53 [16] backbone network with spatial and channel attention mechanisms, enhancing feature extraction from SAR images.

(2) To improve information integration during feature fusion, a progressive cross-fusion mechanism (PCFM) and a feature aggregation module (FAM) are designed to enrich contextual semantic information without loss.

The algorithm is validated using a custom complex scene SAR vehicle dataset. Results demonstrate that, compared to other representative rotating frame detection methods, the proposed approach significantly reduces false alarms and missed detections, thereby improving vehicle position accuracy in SAR images. The rest of this paper is organized as follows: Section 2 details the principles of the proposed algorithm. Section 3 introduces the experimental dataset and environment. Section 4 analyzes the results of the ablation experiments and compares the results with other representative algorithms. Section 5 summarizes the research and outlines potential future research directions.

## 2. Proposed Methods

**Figure 1:**
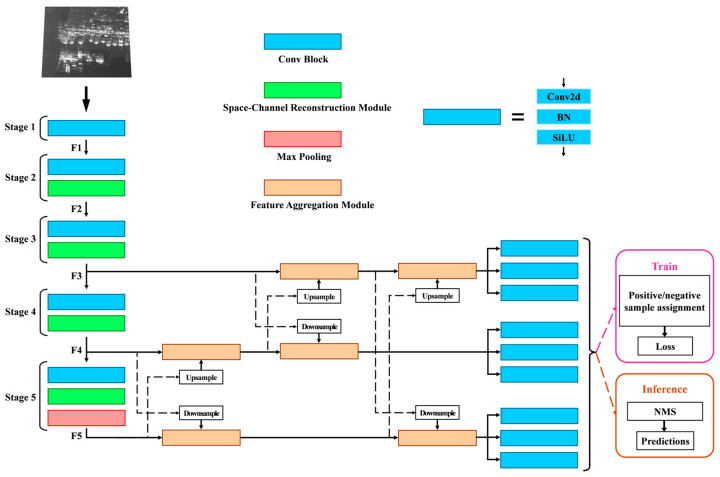
The pipeline of our proposed network. We insert space-channel reconstruction module into the backbone network, design the new progressive cross-fusion mechanism, and insert feature aggregation module into it.

The overall structure of the proposed method is illustrated in Figure 1. The main structures include a feature extraction network, feature fusion network, and detection head network, among which the main innovations include a space-channel reconstruction attention module, progressive cross-fusion mechanism, and feature aggregation module.

Research has shown that CSP-Darknet53 excels in feature extraction for image vision tasks [17]. Consequently, this paper uses the improved CSP-Darknet53 architecture as a feature extraction network. We replace the residual block in CSP-Darknet53 with a spatial-channel reconstruction attention module to improve the feature extraction ability of small-size targets in SAR images. The feature fusion network employs a progressive cross-fusion mechanism specifically designed for SAR image characteristics. This mechanism facilitates bidirectional cross-fusion between deep and shallow features, enhancing information exchange across different feature maps and improving multi-scale retrieval capabilities. The feature aggregation module, constructed with multiple convolution kernels, expands the network’s receptive field, thereby boosting its ability to extract vehicle target features. Additionally, the rotating box detection head simultaneously outputs category information and bounding box regression data. A joint loss function is utilized to optimize the training process, further enhancing vehicle localization accuracy.

In the following, the key points of the SCRM, PCFM, FAM and detecting head are described in detail.

### 2.1. SCRM

**Figure 2:**
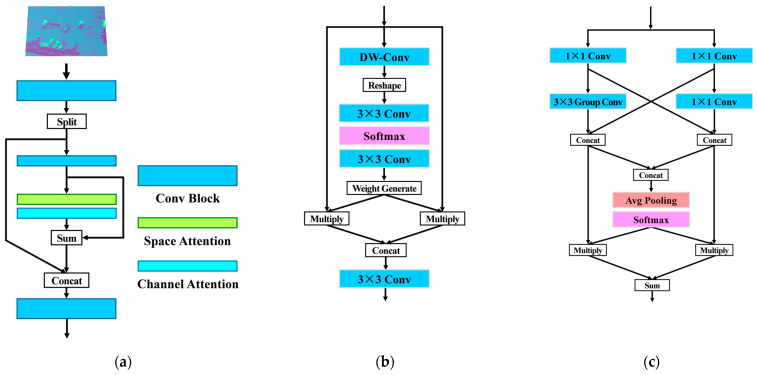
Space-channel reconstruction module. (**a**) The overall structure of the space-channel reconstruction module. (**b**) Spatial attention. (**c**) Channel attention.

Current attention mechanisms often fall short for small-sized objects like vehicles, failing to effectively extract useful information. To address this, there is a need to redesign space and channel attention mechanisms specifically for vehicles in complex scenes. In SAR images, vehicles appear as bright spots with limited contour and texture information, making significant feature extraction challenging with conventional backbone networks. To tackle these issues, this paper introduces a novel attention mechanism, the space-channel reconstruction module (SCRM), as depicted in Figure 2a. The SCRM module integrates spatial and channel attention mechanisms, which are embedded within a residual structure. This approach enhances the vehicle’s salience by capturing contextual semantic information and multi-channel data while suppressing background noise. Figure 2b and Figure 2c illustrate the spatial and channel attention mechanisms, respectively.

Spatial features are crucial for the accuracy of bounding box and angle regression, and the spatial attention mechanism enhances spatial feature extraction. By focusing on the vehicle’s position, spatial attention helps the network emphasize the vehicle’s features while minimizing background information. In the spatial attention mechanism, a non-shared convolution kernel approach is proposed. Unlike common convolution kernels, which share parameters across different positions, non-shared kernels adapt parameters at various positions, allowing for more effective vehicle–background distinction. Specifically, a 3 × 3 convolution kernel is applied in a grouped convolution on the input feature map *X*, with the number of groups equal to the number of input channels to ensure channel information remains isolated. This operation generates nine feature maps of the same size as the input. These nine maps are then deformed into one, expanding the original feature map size by nine times. A subsequent 3 × 3 convolution with a stride of 3 produces a final feature map of the same size as the input, effectively applying different convolution kernels at various positions to achieve varying degrees of focus. The non-shared convolution kernel mechanism produces an intermediate feature layer, which is then activated using the SoftMax function and passing the threshold filter to create two weight layers. Weights exceeding the threshold are set to 1 to enhance high-intensity information *W*_1_, while weights below the threshold are set to 0 to inhibit low-intensity information *W*_2_. This process is outlined in Formula (1), where *X* represents the input to the spatial attention mechanism.

(*W*_1_, *W*_2_) = Gate(Softmax(Conv(Reshape(Conv(*X*))))) (1)

Next, weighted operations and information aggregation are performed. The weights *W*_1_ and *W*_2_ are multiplied with the input feature map and then concatenated. A subsequent convolution operation reduces the number of channels, resulting in an output with the same shape as the input. This process is described by Formula (2), where *Y* represents the output of the spatial attention mechanism.

*Y* = Conv(Concat(*X*⊙*W*_1_, *X*⊙*W*_2_)) (2)

The spatial attention mechanism focuses on extracting information from each channel of the feature map, while the channel attention mechanism operates across different channels, applying weighting operations to extract meaningful information from various feature layers. Different feature layers contain varying types of information, with some being rich and others being redundant. Channel attention helps suppress redundant information and enhance the richness of the channel information. In the designed channel attention mechanism, the input feature map *X* is divided into two channels: left *X*_1_ and right *X*_2_. Both channels undergo convolution to reduce their dimensionality and concentrate channel-specific information. The left channel *X*_1_ is processed through a high-performance convolution module, while the right channel *X*_2_ is processed through a high-efficiency convolution module. The high-performance module utilizes 3 × 3 convolutions and grouped convolutions for robust information extraction, while the high-efficiency module employs two 1 × 1 convolutions to achieve extraction with a lower parameter count. The intermediate results from these modules are concatenated with the outputs from the other channel, resulting in two feature maps, *X*_1′_ and *X*_2′_, which represent the extracted channel information. Subsequently, global pooling and the SoftMax function are applied to generate a weight matrix for the two feature maps, with each weight corresponding to a specific feature layer. The feature maps are multiplied by the weight matrix and then summed to produce the final output, facilitating the activation and fusion of information from different feature layers. The specific process is detailed in Formulas (3)–(5).

*X*_1′_ = Concat(Conv(Conv(*X*_1_), Conv(*X*_2_))) (3)

*X*_2′_ = Concat(Conv(*X*_1_), Conv(Conv(*X*_2_))) (4)

*Y* = (*X*_1′_ + *X*_2′_)·Pool(Softmax(Concat(*X*_1′_, *X*_2′_))) (5)

Spatial attention and channel attention are connected in series via a residual structure. This design enhances the stability of the module, prevents gradient disappearance, and ensures effective feature extraction.

### 2.2. PCFM

**Figure 3:**
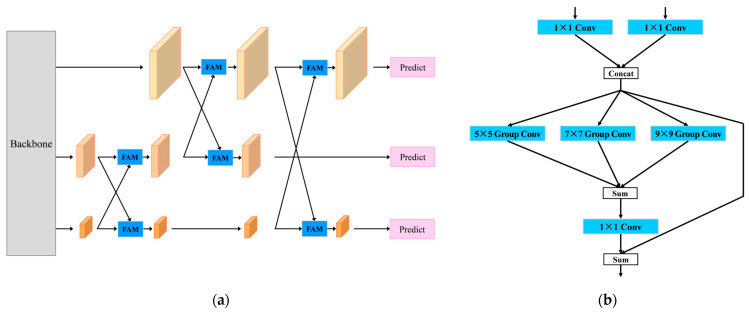
Overall structure of the progressive cross-fusion mechanism (PCFM). (**a**) The structure of the PCFM. (**b**) The structure of the FAM.

Feature fusion enhances communication between different feature layers, improving the positioning accuracy of small objects. Ensuring sufficient information exchange while preserving the original data is a critical consideration. Existing fusion mechanisms often fail to significantly boost vehicle retrieval performance. To address this, we propose a progressive cross fusion mechanism (PCFM), with the overall structure being depicted in Figure 3a. This mechanism not only innovates feature fusion structure but also incorporates a feature aggregation module (FAM) for effective feature map integration, as illustrated in Figure 3b. The FAM is located at the intersection of arrows when two feature maps are fused in the PCFM.

The PCFM enables the progressive fusion of feature layers extracted by the backbone network. Since the semantic information in feature maps varies between layers, particularly between adjacent and non-adjacent layers, the fusion process begins by integrating the lower-resolution, high-abstract maps *F*4 and *F*5. These maps are adjusted to the same shape through upsampling, downsampling, and convolution. The FAM then merges the information from these maps, resulting in two feature maps that combine the information of adjacent layers, with no change in shape before and after fusion. Similarly, the same fusion process is applied to the shallower feature maps *F*3 and *F*4. Subsequently, the FAM is employed after the deeper upsampling, downsampling, and convolution of the non-adjacent layers *F*3 and *F*5, enabling information fusion between these non-adjacent feature layers. This approach enhances information interactions across different feature layers while minimizing information loss during fusion, thereby improving the quality of the feature maps fed into the detection head. The operation is summarized in Formula (6), where *F*3, *F*4, and *F*5 are the original feature maps, *F*3′, *F*4′, and *F*5′ are intermediate feature maps, and *F*3″, *F*4″, and *F*5″ are the final output maps of the PCFM. The arrows denote the fusion process by the FAM module, with the output size being the same as the feature map pointedby the arrow.

*F*4→*F*5 = *F*5′ *F*4←*F*5 = *F*4′ *F*3←*F*4′ = *F*3′
*F*3→*F*4′ = *F*4″
*F*3′←*F*5′ = *F*3″
*F*3′→*F*5′ = *F*5″ (6)

Vehicles exhibit strong backscattering on radar waves due to sharp structures and special materials, leading to significant sparsity in synthetic aperture radar (SAR) images. This sparsity includes amplitude, feature, and parameter sparsity [18]. Strong scattering points are dispersed along the vehicle’s contour, and this scattering information is crucial for detection box regression. However, the dispersion makes it challenging to establish connections between these points. To address this, the feature aggregation module (FAM) employs grouped convolution with three large kernels to enhance the extraction of local information from vehicle images. Grouped convolution helps prevent the mixing of information between different feature layers and reduces the parameter count. The FAM processes two feature maps of the same size by first applying a 1 × 1 convolution to concatenate them. These concatenated maps are then split into three groups for separate convolutions, each with kernel sizes of 5 × 5, 7 × 7, and 9 × 9. The results are integrated through addition and further convolution. This approach not only avoids the mixing of information between the two feature maps and various channels but also minimizes the number of parameters in the module.

### 2.3. Detecting Head

**Figure 4:**
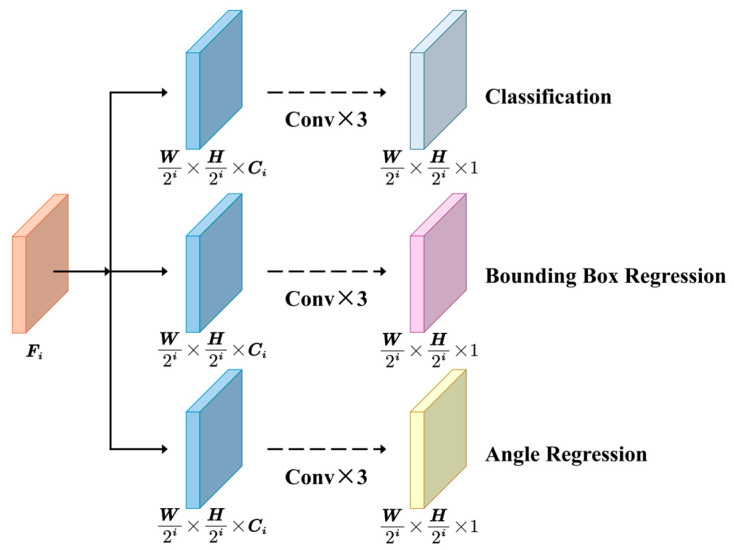
Rotating box vehicle detection head.

To convert feature information into positional data, the rotating box vehicle detection head shown in Figure 4 is utilized. This decoupling head is divided into three components: class prediction, bounding box regression, and bounding box angle regression. Each component consists of two 3 × 3 convolutional layers followed by a 1 × 1 convolutional layer. The class output predicts the probability of vehicle presence, the regression output estimates the bounding box’s position and size, and the angle regression output determines the bounding box’s orientation. Multiple points on the feature map *F*_i_″ ($F_{i}\prime \prime \in R^{W/2^{i}\times H/2^{i}\times C_{i}},\;C_{i}=2^{i+4},\;i=3,\;4,\;5$) produced by the PCFM serve as the centers for the prediction boxes. After convolution, the feature map’s dimensions are adjusted to provide the offset of the prediction box relative to these centers, indicating the distance from the box’s edges. The angle is defined as the rotation of the box relative to the horizontal axis, measured counterclockwise and constrained within the range [0, 90°] degrees.

In addition to the network structure, the performance of the network also relies on the mechanism for matching positive and negative samples. This paper employs TAA [19] as the strategy for allocating positive and negative samples. The process for selecting positive samples is as follows:

(1) Identify prediction boxes whose centers are within the true boxes.
(2) Select the 10 prediction boxes with the highest scores.
(3) Apply IoU filtering to ensure that each predicted box corresponds to only one true box.

The score is computed using Formula (7), where *s* represents the class probability, *u* denotes the IoU between the predicted box and the true box, and α and β are hyperparameters used to balance classification and regression. Consequently, the score *t* for each predicted box is determined.

*t* = *s*^α^ × *u*^β^ (7)

During the training phase, the network’s loss function encompasses both the class loss and bounding box regression loss. The bounding box regression loss comprises IoU loss and boundary loss. The classification loss focuses on distinguishing vehicles from the background, using the binary cross-entropy loss function as specified in Formula (8). In this equation, *y* represents the predicted label, with 0 indicating the background and 1 indicating the vehicle. *p* denotes the output activated by the Sigmoid function, and *N* is the number of predicted samples.

(8) Lcls=−1N∑i=1N[yilog ⁡pi+1−yilog⁡ 1−pi]

The IoU loss, commonly used in rotating frame regression, often performs poorly. Therefore, boundary frame similarity is measured using distance based on a Gaussian distribution, known as ProbIoU [20]. The loss is computed using Formula (9).

(9) $$L_{\mathrm{IoU}}\;=1-ProbIoU$$

Given that vehicle contours in SAR images are often unclear, accurately regressing using only IoU Loss for bounding boxes is challenging. To address this, DFL (Distribution Focal Loss) [21] is incorporated into the loss function, denoted as *L*_DFL_. Consequently, the total loss is computed as shown in Formula (10), where λ_1_, λ_2_, and λ_3_ represent the weights for each component of the loss.

*Loss* = λ_1_*L*_cls_ + λ_2_*L*_IoU_ + λ_3_*L*_DFL_ (10)

## 3. Experimental Design

### 3.1. Datasets Description

This study utilizes two SAR vehicle datasets: the SAR vehicle dataset we made and Mix MSTAR [22].

The SAR vehicle dataset we made is made up of SIVED [23]; the annotation of [24] and the pure background SAR images were collected. This dataset utilizes data sources such as FARAD, MiniSAR, and MSTAR, which include multiple polarization modes and resolutions of 0.1 m × 0.1 m and 0.3 m × 0.3 m, representing commonly used high-resolution land scene SAR data. The resulting dataset comprises 1826 SAR images of size 512 × 512, containing a total of 18,858 vehicle instances. The scenes cover urban areas with complex scattering echoes, dense forested regions, and open areas. For training, the images were randomly divided into training and test sets with an 8:1 ratio (1624 images for training and 203 images for testing).

Mix MSTAR is a dataset with SAR images annotated for vehicle rotation boxes, combining SAR vehicle slices with cluttered backgrounds at the pixel level to produce 100 high-resolution images. This dataset includes 5392 vehicle instances across 20 categories and features backgrounds like woods, grasslands, urban buildings, and crowded parking lots. Out of the one hundred images, thirty-four are merged into four large-format SAR images used as the test set. In generalization experiments, the model trained on the SAR vehicle dataset was evaluated using the test set that has been cropped to 625 512 × 512 images slices, without considering vehicle types. During the testing phase, all categories within the dataset are classified as “vehicles”, as this paper focuses solely on detection.

**Figure 5:**
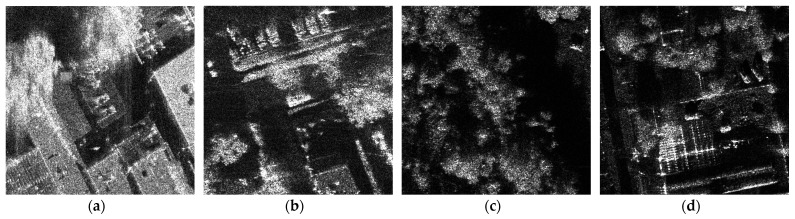
Partial samples of self-made dataset and Mix MSTAR. (**a**,**b**) are from the self-made dataset, and (**c**,**d**) are from Mix MSTAR.

Figure 5 shows partial samples of the self-made dataset and Mix MSTAR: graphs (a) and (b) are from the self-made dataset, while graphs (c) and (d) are from Mix MSTAR. In Figure 5a,c,d, the presence of buildings and vegetation creates strong interference backgrounds, which can lead to false alarms. In Figure 5b, the trees cause partial occlusion of the vehicles, making them prone to missed detections.

### 3.2. Evaluation Indicators

To facilitate the analysis of network performance, this paper uses precision, recall, F1-score, and average precision (AP) as evaluation indicators for different algorithms. Furthermore, we employed floating point operations (FLOPs) and the number of parameters (Params) as key metrics to evaluate both the time complexity and space complexity of our model.

Precision measures the proportion of true positive predictions among all predictions made by the network, while recall indicates the proportion of actual true positives that the network successfully identifies. These metrics are calculated using Formulas (11) and (12), where TP denotes the number of vehicles correctly identified by the algorithm, FP represents the number of vehicles incorrectly classified, and FN refers to the number of vehicles not identified by the algorithm. Higher precision indicates fewer false detections, while higher recall signifies fewer missed detections. The precision and recall indicators are formulated as follows:

(11) $$Precision=\frac{TP}{TP+FP}$$

(12) $$Recall=\frac{TP}{TP+FN}$$

The F1-score is a metric that combines precision and recall, with a value range of [0, 1]. A higher F1-score indicates better overall performance of the algorithm and a more balanced trade-off between precision and recall. It is calculated using the following formula:

(13) $$F1-score=\frac{2\times Precision\times Recall}{Precision+Recall}$$

During prediction, the confidence threshold influences the changes in precision and recall rates. The PR curve is plotted with recall on the *x*-axis and precision on the *y*-axis, and AP is represented by the area under the PR curve and above the coordinate axis. The AP indicator is formulated as follows:

(14) $$AP=\int_{0}^{1}P\left(R\right)dR$$

In addition, frames per second (FPS) is used to measure the speed of detection algorithms. A higher FPS indicates a faster-running algorithm. FPS is defined as follows:

(15) $$FPS=\frac{1}{T}$$

where *T* represents the average inference time required to process a single image.

### 3.3. Experimental Environment and Parameter Setting

To verify the performance of the proposed algorithm, we use the dataset described in Section A and evaluate it based on the metrics introduced in Section B. The experiments are conducted on an Ubuntu operating system with Python 3.10.0 and Pytorch 2.1.2. Training is performed on an NVIDIA GeForce RTX 3090 graphics card. We configure the experiment with a batch size of 16, an initial learning rate of 0.001, and a momentum of 0.957. At the beginning of training, 150 rounds of warm-up iterations were carried out, the warm-up learning rate was set to 0.0005, and the warm-up learning momentum was set to 0.8. After that, 200 rounds of training were carried out, and a total of 350 rounds of iterative training were carried out. Some hyperparameters used in the experiments are listed in Table 1.

## 4. Results

### 4.1. Ablation Experiments

A series of ablation experiments were conducted to assess the effectiveness of the SCRM and the PCFM proposed in this study by incrementally integrating each proposed component into the baseline network. Detailed results of these experiments are presented in Table 2, where the maximum value of each indicator has been highlighted in bold. For these experiments, we utilized the CSPDarkNet backbone network and the PAFPN [25] feature fusion network as our baseline in this study. The proposed method involved gradually incorporated three key components into the baseline: the space-channel reconstruction module, the progressive cross-fusion mechanism, and the feature aggregation module. The dataset created in this study was employed for performance evaluation.

As shown in Table 2, each module we proposed contributes to enhancing the performance of the overall network compared with the baseline network. Specifically, the SCRM significantly improves vehicle detection accuracy, with notable advancements across three key performance metrics. The PCFM structure without the FAM reduces the vehicle detection accuracy slightly, demonstrating that the structure alone is not conducive to the improvement of performance. However, once the FAM is integrated, the network’s detection capabilities are substantially enhanced. When all three components are combined, the overall network leads to significant degrees of improvement in metrics such as precision, recall, and AP. Specifically, there are increases of 1.6%, 2.73%, and 1.53%, respectively, demonstrating the higher accuracy of the proposed modules. These results validate the effectiveness of FRA-Net and highlight the synergistic benefits of the combined approach. It is important to note that, while the integration of the PCFM and FAM modules into the network alongside the SCRM module resulted in a decline in precision and F1-score, we believe that AP is the most critical metric for evaluation. Consequently, we conclude that the combination of the three models yields the best overall performance.

### 4.2. Comparative Experiment

In this section, we compare FRA-Net with other methods for detecting rotatable bounding boxes on the dataset created in this study to evaluate the effectiveness of the proposed SAR vehicle localization method. We employ the metrics introduced above as benchmarks for performance assessment. Our method is compared with several advanced rotatable bounding boxes object detection methods under the training conditions described above.

A brief overview of these methods is provided below. Rotated Faster R-CNN [5] is a two-stage object detection model that integrates the region of proposal and pooling layer extraction. It enhances classification and detecting accuracy through the addition of angle regression, which facilitates multi-task learning. Gliding Vertex [26] is also a regression strategy, including rotatable bounding boxes and the sliding of the vertex of a horizontal box to represent the rotated box. This approach avoids the confusion that can arise from directly regressing the vertices of the rotating box. KLD [27] is a novel strategy for rotating bounding box, which takes the Kullback–Leibler divergence between the two-dimensional Gaussian distributions of rotating bounding boxes as a regression loss. It is incorporated into the Rotate RetinaNet framework during the experiment. Like KLD, GWD [28] models the rotated bounding box as a Gaussian distribution. However, it uses a loss function based on the Gaussian Wasserstein distance to calculate the regression loss, offering a different approach to bounding box representation. S^2^A-Net [29] introduces a feature alignment module and a direction regression module into the detection head, which facilitate the generation of high-quality anchors and improve the balance between classification and regression accuracy. In order to achieve high precision orientation, Oriented RepPoints [30] employs an adaptive point learning approach in the shared head. During training, it learns to select high-quality points samples, which aids in achieving high-precision object localization scores and classification confidence. KFIoU [31] models the rotating box as a Gaussian distribution and utilizes Kalman filtering to approximate the SkewIoU loss. This approach is particularly well suited for models based on gradient training, as it provides a more accurate representation of the localization loss.

We conducted a quantitative analysis of various rotatable bounding boxes detection methods introduced above using the dataset created in this study, and the specific detection results are presented in Table 3. The table indicates that our proposed method achieved the highest values in terms of precision, recall, and AP, with respective scores of 98.24%, 99.55%, and 99.27%. Specifically, the AP achieved by our proposed method was 2.04% higher than the lowest performance method, GWD, and 0.39% higher than the highest performance method, Oriented RepPoints. Additionally, the proposed method also leads in precision and recall rates, with scores 0.27% higher than the best method, KLD, and 0.45% higher than the highest recall method, Oriented RepPoints. Importantly, the proposed method maintains a balance between precision and recall, achieving the highest F1-score. Our method effectively minimizes missed detections while controlling false alarms. In comparison, the Oriented RepPoints method has the second highest recall, but the precision is too low, impacting its overall detecting accuracy.

In addition, the values of Params and FLOPs in the table demonstrate that our proposed method achieves the lowest values, giving it a clear advantage over other rotating frame target detection methods in terms of both space and time complexity. Moreover, our method also stands out in terms of detection speed, with the highest FPS, at 22.8.

**Figure 6:**
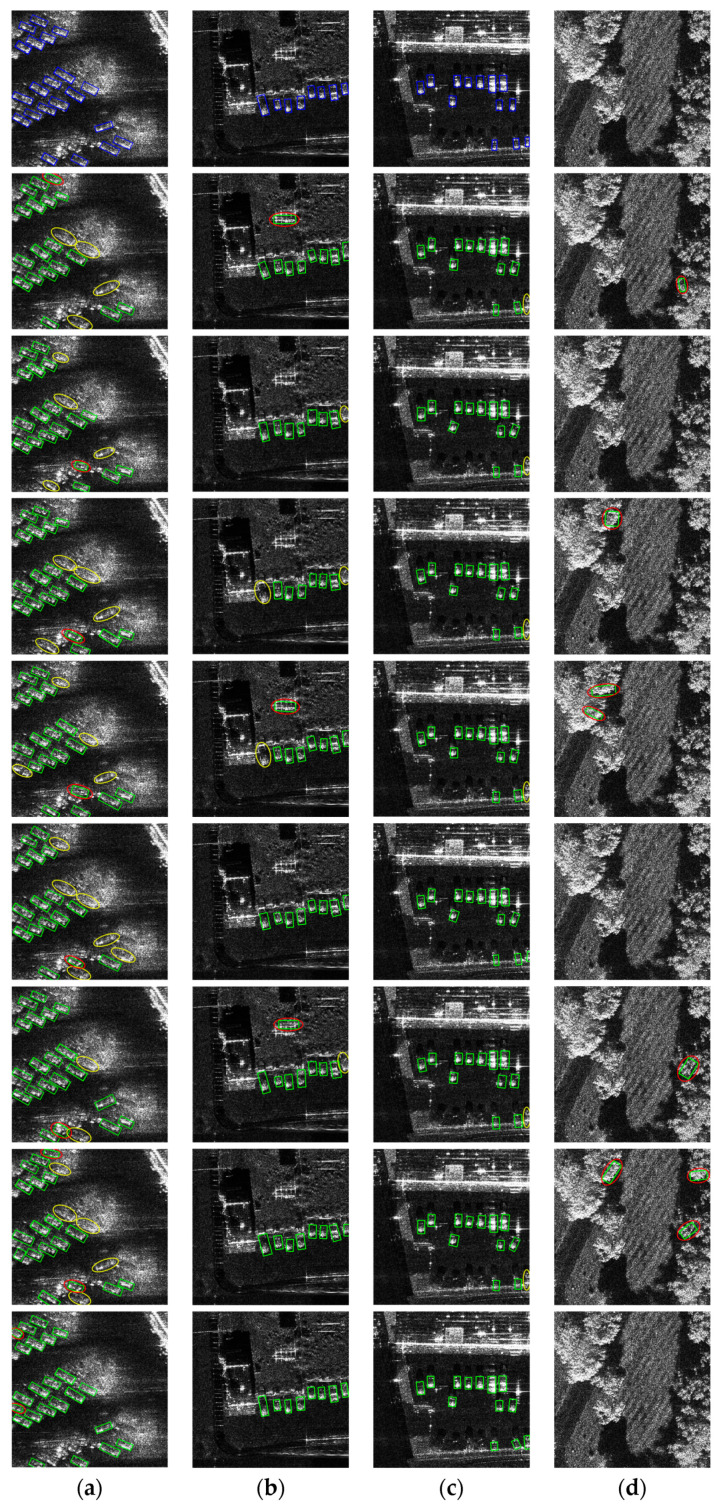
Comparison of experimental results of different methods on our SAR vehicle dataset. (**a**) has vegetation interference; (**b**,**c**) have strong scattering from buildings; (**d**) is pure background. The blue boxes represent ground truths, while the green boxes denote detected vehicles. False alarms are circled by red ovals, while missing vehicles are circled by yellow ovals. The first row indicates the ground truth, and the second row to the ninth row indicate the detection result of Rotated Faster R-CNN, Gliding Vertex, KLD, GWD, S^2^A-Net, Oriented RepPoints, KFIoU, and our method.

We also conducted a qualitative analysis on our SAR vehicle dataset, and the visualization results are presented in Figure 6. The figure shows visualization results in four different scenarios of the SAR vehicle datasetfrom Rotated Faster R-CNN, Gliding Vertex, KLD, GWD, S^2^A-Net, Oriented RepPoints, KFIoU, and our method, in which blue boxes indicate the real targets in the images, green boxes indicate the targets detected by the network, yellow ellipses indicate the missed targets, and red ellipses indicate the mis-detected targets. In the first image, the vehicle slice images are severely obstructed by vegetation; in the second and third images, there is strong interference from buildings; and the fourth image is a pure background image with a lot of disturbing information. The task of locating vehicles in these images is quite challenging.

For the first image, the method proposed in this paper successfully detected all vehicles without any missed detections, whereas other methods failed to detect some vehicles, especially in areas heavily obstructed by vegetation in the center of the image. Additionally, in regions with strong echoes at the bottom of the image, methods such as Gliding Vertex, KLD, and GWD produced false detections. For the second image, the proposed method identified all vehicles accurately without false detections, while other methods either misclassified strongly scattered buildings as vehicles or missed vehicles with weaker scattering intensity. For the third image, all methods except our method produce a false vehicle alarm in the lower right corner of the image. For the fourth image, the proposed method did not produce any false detections, whereas Rotated Faster R-CNN, KLD, GWD, Oriented RepPoints, and KFIoU methods incorrectly identified strong echo vegetation as vehicles. Finally, comparing the detection results with the ground truth boxes, it is evident that all methods performed worse in terms of rotation box localization and orientation accuracy compared to the method proposed in this paper. Overall, the other methods exhibit severe missed and false detections in complex scenarios, while the proposed method excels in minimizing these issues and achieves the highest vehicle detecting accuracy. Therefore, the algorithm proposed in this paper can not only improve the detection performance of targets in complex scenes, but can also enhance the localization and orientation performance for vehicles.

### 4.3. Generalization Experiment

In addition to evaluating algorithm performance, we conducted generalization experiments. We used the best models from each method trained on the self-made dataset in Section 4.3, and we applied them to the cropped Mix MSTAR test set described in Section 3.1. The models were put into inference mode to obtain the test results. For evaluation, we used precision, recall, F1-score, and AP as the metrics.

Based on the results from Table 3 and Table 4, it is evident that the performance of all methods is significantly diminished. The discrepancy between the distribution characteristics of the training and validation datasets substantially limits network performance, highlighting that network generalization remains a challenging issue. Nevertheless, our method exhibits considerable improvement in generalization and achieves the highest AP value compared to others. Additionally, our approach maintains a balanced precision and recall, with the F1-score reaching its maximum. The Oriented RepPoints method for remote sensing image rotation frame detection continues to perform robustly, attaining an AP value of 68.12%, while other methods fall below 60%.

## 5. Conclusions

This paper proposes a vehicle localization method for SAR images based on feature reconstruction and aggregation. The proposed method comprises three key components: the SCRM, PCFM, and FAM. The SCRM introduces a novel attention mechanism designed for spatial features and channel features, respectively, and effectively captures contextual semantic information and multi-channel data from SAR images, thereby enhancing vehicle information and reducing background noise. The PCFM is a feature fusion mechanism, and the FAM is a specific feature fusion module within it. Together, they will expand the receptive field region and enhance contextual information to improve vehicle feature representation in SAR images. The integration of these three components into an SAR image vehicle detection network addresses the limitations of the existing methods, such as low vehicle detecting accuracy and high rates of false alarms and missed detections. By utilizing rotating box predictions, the proposed method significantly enhances vehicle localization accuracy in SAR images. Experimental results demonstrate that the proposed approach achieves high precision in vehicle localization and orientation in both complex urban and open areas.

Additionally, the SCRM introduced in this paper is applicable to any CNN-based detector, and the proposed PCFM improves feature fusion effectiveness, making it adaptable to other object detection models. However, due to dataset’s certain limitations, the proposed method currently focuses solely on vehicle localization and does not address vehicle classification. In future research, we plan to explore methods for vehicle detection and classification in intricate SAR images with more confusing objects.

## Figures and Tables

**Table 1:** Network hyperparameters.

Parameter	Value
initial learning rate	0.001
final learning rate	0.00001
momentum	0.957
warm-up epochs	150
warm-up bias learning rate	0.0005
warm-up momentum	0.8
batch size	16
epochs	350

**Table 2:** Experimental results of ablation experiments.

	SCRM	PCFM	FAM	Precision	Recall	F1-Score	AP
Baseline	-	-	-	96.64%	96.82%	96.73%	97.74%
FRA-Net	✓	-	-	**98.94** **%**	98.86%	**98.90** **%**	98.80%
	-	✓	-	94.97%	94.79%	94.88%	97.36%
	-	✓	✓	95.74%	96.97%	96.35%	98.29%
	✓	✓	✓	98.24%	**99.55** **%**	98.89%	**99.27** **%**

“-”: The module is not in use. “✓”: The module has been added. Bold: Highest value of each column.

**Table 3:** Experimental results of different models.

Method	Precision	Recall	F1-Score	AP	Params (M)	FLOPs (G)	FPS
Rotated Faster R-CNN [5]	96.80%	98.96%	97.87%	98.60%	41.12	78.12	11.2
Gliding Vertex [26]	96.74%	98.44%	97.58%	98.03%	41.13	78.12	10.6
KLD [27]	97.97%	98.48%	98.22%	98.16%	36.13	81.87	10.7
GWD [28]	96.06%	98.15%	97.09%	97.23%	36.13	81.87	11.2
S^2^A-Net [29]	96.27%	97.96%	97.11%	97.35%	38.54	76.64	9.8
Oriented RepPoints [30]	96.16%	99.10%	97.61%	98.88%	36.60	75.88	10.2
KFIoU [31]	96.03%	98.53%	97.26%	97.97%	41.58	128.40	7.5
Our method	**98.24** **%**	**99.55** **%**	**98.89** **%**	**99.27** **%**	**11.03**	**23.90**	**22.8**

Bold: Highest value of each column.

**Table 4:** Migration experiments for the Mix MSTAR dataset.

Method	Precision	Recall	F1-Score	AP
Rotated Faster R-CNN [5]	77.98%	58.64%	66.94%	51.65%
Gliding Vertex [26]	**79.19** **%**	48.46%	60.13%	43.92%
KLD [27]	63.47%	61.52%	62.48%	55.57%
GWD [28]	42.29%	64.30%	51.02%	51.09%
S^2^A-Net [29]	67.39%	57.44%	62.02%	49.57%
Oriented RepPoints [30]	60.05%	76.66%	67.35%	68.12%
KFIoU [31]	40.73%	61.28%	48.94%	52.98%
Our method	65.03%	**73.04** **%**	**6** **8.** **80** **%**	**68.31** **%**

Bold: Highest value of each column.

## Data Availability

The data of the experimental images used to support the findings of this research are available from the corresponding author upon reasonable request. The data are not publicly available due to privacy.
